# Telemonitoring of Crohn’s Disease and Ulcerative Colitis (TECCU): Cost-Effectiveness Analysis

**DOI:** 10.2196/15505

**Published:** 2019-09-13

**Authors:** Javier Del Hoyo, Pilar Nos, Guillermo Bastida, Raquel Faubel, Diana Muñoz, Alejandro Garrido-Marín, Elena Valero-Pérez, Sergio Bejar-Serrano, Mariam Aguas

**Affiliations:** 1 Gastroenterology Department La Fe University and Polytechnic Hospital Valencia Spain; 2 Networked Biomedical Research Center for Hepatic and Digestive Diseases Valencia Spain; 3 Health Research Institute La Fe Valencia Spain; 4 Physiotherapy Department University of Valencia Valencia Spain; 5 Joint Research Unit in Biomedical Engineering-eRPSS Health Research Institute La Fe–Polytechnic University of Valencia Valencia Spain

**Keywords:** telemedicine, eHealth, cost-effectiveness, inflammatory bowel diseases, Crohn disease, colitis, ulcerative

## Abstract

**Background:**

Although electronic health interventions are considered safe and efficient, evidence regarding the cost-effectiveness of telemonitoring in inflammatory bowel disease is lacking.

**Objective:**

We aimed to evaluate the cost-effectiveness and cost-utility of the *Telemonitorización de la Enfermedad de Crohn y Colitis Ulcerosa* (Telemonitoring of Crohn’s Disease and Ulcerative Colitis [TECCU]) Web platform (G_TECCU intervention group) for telemonitoring complex inflammatory bowel disease, compared with standard care (G_control) and nurse-assisted telephone care (G_NT intervention group).

**Methods:**

We analyzed cost-effectiveness from a societal perspective by comparing the 3 follow-up methods used in a previous 24-week randomized controlled trial, conducted at a tertiary university hospital in Spain. Patients with inflammatory bowel disease who initiated immunosuppressants or biologic agents, or both, to control inflammatory activity were recruited consecutively. Data on the effects on disease activity (using clinical indexes) and quality-adjusted life-years (using the EuroQol 5 dimensions questionnaire) were collected. We calculated the costs of health care, equipment, and patients’ productivity and social activity impairment. We compared the mean costs per patient, utilities, and bootstrapped differences.

**Results:**

We included 63 patients (21 patients per group). TECCU saved €1005 (US $1100) per additional patient in remission compared with G_control (95% CI €–13,518 to 3137; US $–14,798 to 3434), with a 79.96% probability of being more effective at lower costs. Compared with G_NT, TECCU saved €2250 (US $2463) per additional patient in remission (95% CI €–15,363 to 11,086; US $–16,817 to 12,135), and G_NT saved €538 (US $589) compared with G_control (95% CI €–6475 to 5303; US $–7088 to 5805). G_TECCU and G_NT showed an 84% and 67% probability, respectively, of producing a cost saving per additional quality-adjusted life-year (QALY) compared with G_control, considering those simulations that involved negative incremental QALYs as well.

**Conclusions:**

There is a high probability that the TECCU Web platform is more cost-effective than standard and telephone care in the short term. Further research considering larger cohorts and longer time horizons is required.

**Trial Registration:**

ClinicalTrials.gov NCT02943538; https://clinicaltrials.gov/ct2/show/NCT02943538 (http://www. webcitation.org/746CRRtDN)

## Introduction

### Background

Health systems are facing problems of financial sustainability, and the burden and health care costs associated with the management of inflammatory bowel disease (IBD) continue to rise [1,2]. IBD is one of the most expensive gastrointestinal conditions [3]. In this context, interest in electronic health (eHealth) interventions as a potential means to improve health care services at a lower cost has grown in recent years, especially for the management of chronic diseases such as IBD [4,5].

Unlike other chronic pathologies, IBD mainly affects young individuals in their optimal period of personal and professional development. As such, IBD is related to high levels of school absenteeism and work disability [6], interference in social activities, and impaired health-related quality of life [7]. Therefore, IBD has a significant medical, social, and financial impact, which accounts for direct and indirect costs to both health care systems and society. In fact, although the estimated indirect costs differ between countries depending on their health policies, they are an important percentage of the IBD-related economic burden. A recent report estimated them to account for 46.5% of the total IBD-related costs in Spain [8].

Web-based telemonitoring systems applied to IBD are safe and feasible not only for adults but also for adolescents [9-11], and empowerment through these systems reduces outpatient visits and hospital admissions, with potential cost savings [12-14]. However, even if eHealth is considered a promising option to improve the quality of care while reducing costs, its efficacy in terms of disease outcomes has not been consistent across studies [10-13,15,16], with high attrition rates despite the continued adaptation of Web platforms and the evolution of mobile health over the last decade.

These systems aim to shift the emphasis from hospital and personal visits toward remote encounters, although it is still necessary to determine whether such telemonitoring systems actually decrease direct and indirect costs [12,13,16]. While cost savings have been attributed to such systems in the IBD setting, these are almost exclusively related to direct costs [12,14]. Indeed, these studies did not consider either the costs associated with the implementation and maintenance of the remote monitoring systems or indirect costs. Thus, evidence regarding the cost-effectiveness and cost-utility of telemonitoring in IBD is lacking [17], without economic data available to evaluate its use in patients with complex IBD, hindering the long-term implementation of these systems by health care services [18].

### Objective

Our research group developed a Web-based telemanagement program known as *T* *elemonitorización de la Enfermedad de Crohn y Colitis Ulcerosa* (Telemonitoring of Crohn’s Disease and Ulcerative Colitis [TECCU]) [19]. The results of a pilot clinical trial suggested that this remote monitoring system is a safe strategy to improve health outcomes in patients with complex IBD, while reducing the use of health care resources when compared with nurse-assisted telephone care and standard face-to-face visits [20]. Due to the limited knowledge about the efficiency of telemonitoring in IBD and given the absence of health economics data in a non–remission setting, we aimed to evaluate the cost-effectiveness and cost-utility of the Web-based TECCU system for remote management of patients with complex IBD from a societal perspective, comparing telemonitoring versus standard care and telephone care.

## Methods

### Study Design

We performed a cost-effectiveness analysis alongside a previously published 3-arm parallel-group randomized controlled trial, which had been carried out at a referral university hospital in Spain [20]. We evaluated the impact of the TECCU Web-based telemanagement system (G_TECCU intervention group), nurse-assisted telephone care (G_NT intervention group), and standard face-to-face visits (G_control) on the health outcomes and the direct and indirect costs associated with patients with complex IBD, after a 24-week follow-up. This follow-up period allowed us to evaluate the impact of these 3 interventions and the events that occurred during the initiation of therapy with immunosuppressants and biologic agents.

With the aim of analyzing cost-effectiveness and cost-utility from a societal perspective, we used a standard economic evaluation methodology to measure the costs and effects on disease activity and quality of life associated with each of the 3 interventions [21]. First, we determined the categories to be included in both the costs and effects analyses. We included health care, equipment, and productivity and social activities in the costs study, while the effects measured were disease activity and quality of life. Subsequently, to analyze the costs in each category, we measured the number of units of health care and non–health care resources consumed, thereafter calculating the cost in euros (and including the exchange in US $) by multiplying the number of units used by their price weight. Finally, we correlated the cost and effect data to obtain a cost-effectiveness and cost-utility comparison between the 3 follow-up strategies.

### Patient Selection and Setting

Patients were recruited consecutively at the Outpatient Clinic of the IBD Unit at La Fe University and Polytechnic Hospital, Valencia, Spain, or at the Gastroenterology Department if they were admitted for a flare-up. This is a tertiary hospital that serves more than 1500 patients with IBD, and it has 2 specialist IBD nurses, also providing its patients with an email and telephone consultation service. All participants had IBD diagnosed according to internationally accepted criteria at least 6 months prior to their recruitment [22,23]. The inclusion criteria were age 18 years or older; and having initiated therapy with corticosteroids, immunosuppressants, or biologic agents, or a combination of these, due to disease activity. The exclusion criteria were inability to speak and read Spanish; inability to manage a mobile phone or tablet, or the internet, or not having a telephone line; participation in other clinical trials during the inclusion period; having other uncontrolled medical or psychiatric disease; the presence of ileorectal or ileal pouch-anal anastomosis; having received a definitive ileostomy; having associated perianal disease; and being pregnant. All participants provided their written informed consent to participate in the study. Enrollment began in October 2014 and ended in June 2016. The follow-up ended in December 2016. Eligible patients were randomly assigned to 1 of the 3 groups to G_TECCU, G_NT, or G_control in a 1:1:1 ratio. A block randomization method was used via a Web-based tool [19] to generate a random allocation sequence and ensure allocation concealment.

### Interventions

Regardless of the assigned arm, all patients completed 3 face-to-face visits, at baseline, 12 weeks, and 24 weeks, in addition to their routine visits to the IBD clinic, telephone consultations, or Web telemonitoring in accordance with their group assignment. Patients treated with immunosuppressants alone or in combination with biologic agents were monitored every 1 to 2 weeks during the first month, every 2 to 4 weeks between month 1 and month 3, and every 4 weeks from month 3 until the end of the follow-up. Patients treated with biologic agents alone were monitored every 2 to 4 weeks during the entire follow-up period. Patients from all 3 arms who initiated the same drug complied with these follow-up schedules, which differed only in the monitoring method used for the study group they were assigned to. In any of the 3 arms, additional clinical visits were made when necessary if the patient’s evolution so required. The 3 interventions evaluated in this clinical trial have been described previously [19] but we present a summary below.

#### TECCU Web Platform

In the G_TECCU follow-up and monitoring was performed telematically using the TECCU app. The patients used a computer, or an app on a mobile phone or tablet, to connect via the internet and self-complete questionnaires related to their IBD symptoms. Through these questionnaires, they also provided information regarding any possible adverse effects. The health care providers used the information obtained from questionnaires and biological markers to adapt medication and follow-up schedules through an intelligent prioritization system. These changes were communicated through the platform’s messaging system, in combination with telephone calls or in-person visits when necessary.

#### Usual Care Provided at the Inflammatory Bowel Disease Unit

Patients from the G_control received usual care provided by the IBD unit (Outpatient Clinic) based on national and European clinical guidelines [22-24]. Treatment was adjusted at face-to-face visits or via information provided through telephone calls based on the evolution of disease activity. Disease activity was measured using specific indexes and through biological markers.

#### Nursing Care by Telephone

In the G_NT, patients were asked about their health during telephone calls with the nursing staff at the IBD Unit. Periodic telephone assessment was carried out using structured interviews to evaluate the patient’s health status based on the same clinical indexes and biological markers used for the other 2 groups. The interventions depended on the results of the interview, and any changes to medication or the follow-up schedule were established by the nurses with the aid of a physician through alerts and action plans incorporated into the intervention protocol [19].

### Cost Measures

We established 3 major cost categories to perform an economic evaluation from a societal perspective: health care costs, equipment costs, and costs related to patients’ work productivity and impaired social activity. We expressed all costs in euros (including the exchange in US $) and corrected for price inflation in 2016 according to the Spanish consumer price index [25].

#### Health Care Costs

We measured health care resource use on the basis of the number of outpatient visits, telephone calls, emergency visits, hospitalizations, and IBD-related surgeries detected in the hospital registry over the study period. We calculated their associated costs by multiplying the number of services registered by the official regional rates [26]. We also considered telemonitoring contacts through the Web platform in G_TECCU. As there is as yet no rate for telemonitoring contacts in Spain, we calculated this rate by multiplying the mean time spent by health care providers in each telemonitoring contact (8 minutes, based on consultation with nurses and physicians) by the mean cost of their salary per minute (€0.21 [US $0.23]/minute for a nurse and €0.38 [US $0.42]/minute for a physician), using data published by the Spanish National Statistical Institute [27].

#### Equipment Costs

Equipment costs included those related to software development, Web security, and technical support. These equipment costs were incorporated into the health care cost of Web telemonitoring, as the software is a necessary expenditure for this health care initiative.

#### Productivity and Social Activity Costs

To assess the number of hours that patients lost from work and social activities, we used the Work Productivity and Activity Impairment questionnaire [28]. The Spanish version of this questionnaire has been validated in patients with Crohn disease. We measured the number of working hours lost due to absenteeism and presenteeism associated with disease activity at baseline and at 12 and 24 weeks, and we also registered the number of hours lost from the patients’ leisure time in the same periods.

We calculated the cost of absenteeism by multiplying the hours lost (question 2 of the Work Productivity and Activity Impairment questionnaire) by the average hourly wage for age and sex according to the Spanish National Statistical Institute [29]. We estimated lost work hours due to presenteeism by multiplying the percentage impairment while working (question 5) by the hours worked over the past 7 days (question 4), and then calculated the cost similarly to the cost of absenteeism hours. To calculate the cost of leisure time lost, we used the percentage of impairment of social activities (question 6) and data regarding the costs of leisure time based on manuals for economic evaluations previously published in Europe [30]. Due to the limited time horizon, we did not discount costs and we considered the human capital approach to evaluate costs associated with the loss of productivity [31].

In addition, we calculated the cost of absenteeism associated with medical visits, assuming a loss of 3.3 hours per visit, as previously reported for patients with Crohn disease in Spain [32]. We calculated the cost of the time that patients spent on telephone contacts assuming that they were conducted during working hours, because they were made between 8 AM and 3 PM. We considered the cost of leisure time for contacts through the Web telemonitoring platform, assuming that patients accessed this tool out of office hours.

### Effect Measures

The primary outcome used for the cost-effectiveness analysis was the effect of each intervention (G_TECCU, G_NT, and G_control) on the percentage of patients in remission throughout the study. The clinical indexes used for the follow-up in the 3 arms were the Harvey-Bradshaw index for patients with Crohn disease [33], and the Simple Clinical Colitis Activity Index and the partial Mayo score for patients with ulcerative colitis [34,35]. As described in the study protocol [19], we considered remission to be indicated by a Harvey-Bradshaw index score of 4 or less in patients with Crohn disease, and a Simple Clinical Colitis Activity Index and partial Mayo score of 2 or less in patients with UC. We compared the proportion of patients in remission at baseline versus that after 24 weeks.

We also measured the number of quality-adjusted life-years (QALYs) for the cost-utility analysis. To measure quality of life, patients answered the EuroQol 5 dimensions questionnaire (EQ-5D) [36] at baseline and at 24 weeks, from which we calculated QALYs using specific coefficients established for the Spanish population [37]. We also briefly described patients’ perceptions and satisfaction with the care received at baseline (previous standard care) and at 24 weeks (assigned follow-up intervention) by using a previously adapted version of the Client Satisfaction Questionnaire [38] for the study purpose, which we described in more detail elsewhere [20].

### Cost-Effectiveness and Cost-Utility Analyses

We divided the calculated costs by the effects of the 3 interventions over 24 weeks to assess cost-effectiveness (considering the improvement in disease activity) and cost-utility (considering the impact on QALYs). The differences in costs between the 3 interventions divided by the differences in their effects allowed us to obtain the incremental cost-effectiveness ratio (ICER). In decision making, ICERs are more useful when the new intervention is more costly but generates an improved health effect [39].

**Figure 1:**
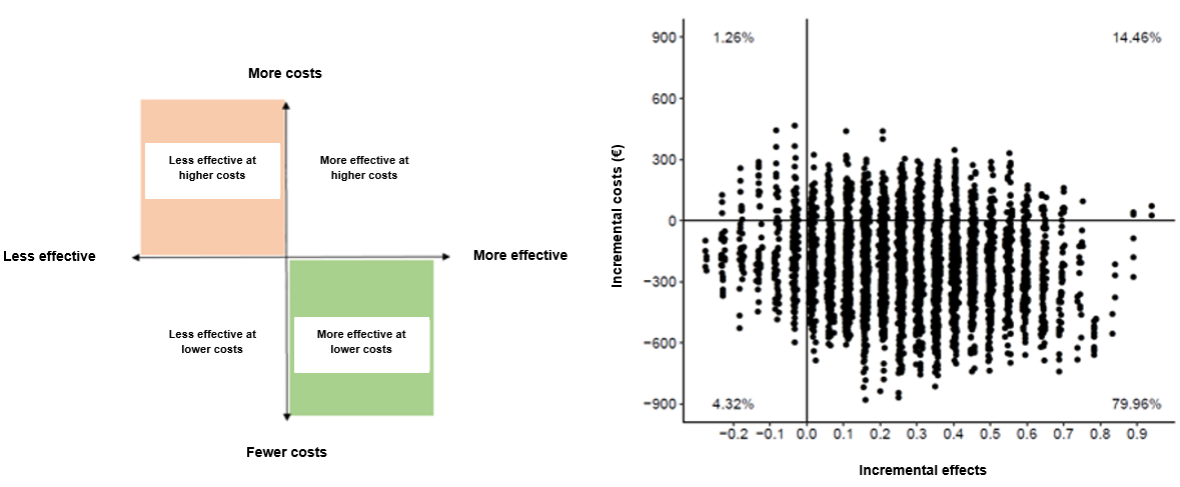
Generic cost-effectiveness plane (left) and an example illustrating the bootstrapped incremental cost-effectiveness ratios plotted (right).

To evaluate the strength of the economic evaluation and the influence of the cost values on the median ICERs calculated, we carried out sensitivity analyses in which we increased and reduced the main cost drivers independently. We also evaluated the uncertainty in the cost-effectiveness and cost-utility analyses. We visually represented the distribution of possible values that the ICERs could acquire as dots plotted on a cost-effectiveness plane (see Figure 1). In this plane, the horizontal axis represents the differences in health outcomes and the vertical axis represents the differences in costs between the 3 interventions compared (G_TECCU vs G_control, G_TECCU vs G_NT, and G_NT vs G_control).

**Figure 2:**
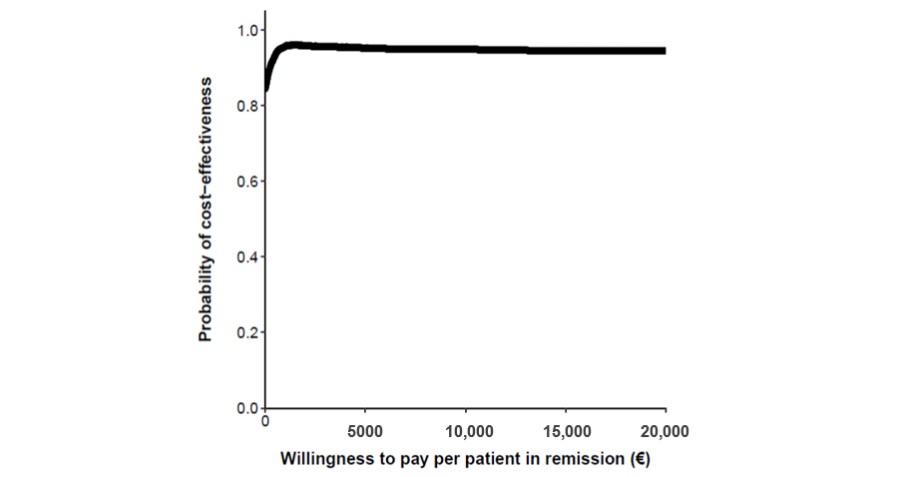
Cost-effectiveness acceptability curve representing the probability that one intervention is cost-effective relative to another, depending on the willingness-to-pay value.

We drew cost-effectiveness acceptability curves to map (on the vertical axis) the evolution of the probability that one intervention is cost-effective compared with another, as a function of the willingness to pay (WTP) for 1 additional unit of effect in a range of €0 to €20,000 (US $0 to 21,893, represented in the horizontal axis; see Figure 2). As part of the sensitivity analysis, we also assessed the influence of alternative costing scenarios on the cost-effectiveness acceptability curve.

### Statistical Analysis

We handled missing observations in costs and effects data using multiple imputation with the Amelia II for R software package version 1 [40]. We imputed the original dataset 5 times and then analyzed each of these 5 datasets separately, subsequently combining the outcomes using Rubin rules. We calculated the differences and statistical uncertainty in the disease activity, QALYs, and costs using nonparametric bootstrap estimations, which consisted of extracting 1000 random samples (n=21 per trial arm) from each of the 5 imputations. For each of these samples, we calculated the incremental costs, incremental effects, and ICERs. We performed these analyses using R version 3.5.1 (R Foundation).

### Ethical Considerations

The study protocol was reviewed and approved by the local independent ethics committee at La Fe University and Polytechnic Hospital, Valencia, Spain; by the regional independent ethics committee (*Comité Ético Autonómico de Estudios Clínicos de Medicamentos y Productos Sanitarios de la Comunitat Valenciana*); and by the Spanish Agency of Medicines and Medical Devices (*Agencia Española de Medicamentos y Productos Sanitarios*). According to the physicians involved in the study, the risks did not outweigh the potential benefits, and each participant provided their informed consent without coercion before inclusion in the study. The randomized controlled trial is registered at ClinicalTrials.gov (NCT02943538; Multimedia Appendix 1 [41]).

## Results

### Patient Characteristics

We invited a total of 68 patients with complex IBD to participate in this study, of whom 3 (4%) declined to participate, as they did not have internet access at their home, and 2 (3%) did not meet the inclusion criteria. The remaining 63 eligible patients were randomly assigned to the 3 groups (21 patients in each group). During the study period, 3 patients in G_TECCU did not adhere to the study protocol, while the remaining 18 patients (86%) showed good adherence, as compared with 19 patients (90%) in G_control and 20 patients (95%) in G_NT. The mean age of the patients was 39.50 (SD 12.06) years, and 52% (33/63) of the patients were women. At baseline, the patients in G_TECCU and G_NT had higher fecal calprotectin levels and satisfaction scores with previous in-person care, with a lower adherence to medication in G_NT according to the validated 4-item Morisky-Green questionnaire [42], which is easy to answer and has been previously used to evaluate adherence to telemonitoring in IBD patients [15]. The other baseline characteristics did not differ markedly between the groups, although quality-of-life scores were slightly higher in G_TECCU and G_NT (Table 1).

**Table 1:** Patients’ baseline characteristics.

Characteristics		Control group (n=21)	Telephone care group (n=21)	TECCU^a^ care group (n=21)
Age (years), median (range)		39.31 (22-61)	40.91 (24-60)	41.32 (19-66)
**Sex, n (%)**				
	Male	12 (57)	12 (57)	9 (43)
	Female	9 (43)	9 (43)	12 (57)
**Education, n (%)**				
	Primary	4 (19)	4 (19)	5 (23)
	Secondary	9 (43)	6 (29)	6 (29)
	University	8 (38)	11 (52)	10 (48)
**Disease profile, n (%)**				
	Crohn disease	14 (67)	13 (62)	13 (62)
	Ulcerative colitis	7 (33)	8 (38)	8 (38)
Median time since diagnosis, months (range)		123.32 (6-427)	108.27 (7-452)	146.72 (7-424)
**Treatment, n (%)**				
	Immunomodulators	10 (48)	10 (48)	9 (43)
	Biologic monotherapy	4 (19)	4 (19)	4 (19)
	Combination therapy	6 (29)	5 (24)	6 (29)
	Corticosteroids	1 (5)	2 (10)	2 (10)
Calprotectin (μg/g), median (IQR^b^)		330 (103-617)	526 (115-1724)	490 (23-2016)
**Quality-of-life scores, median (IQR)**				
	IBDQ-9^c^	38.50 (33.25-46.75)	37.50 (28.75-46.25)	42.00 (33.75-47.50)
	EQ-5D^d^	0.82 (0.75-0.91)	0.83 (0.71-0.92)	0.83 (0.58-0.91)
	Visual analog scale (%)	60.50 (50-85)	62.50 (50-80)	60 (40-90)
Medication adherence, n (%)		14 (67)	7 (33)	12 (57)
**Work Productivity and Activity Impairment questionnaire responses**				
	Not working, n (%)	8 (38)	7 (33)	5 (24)
	Work hours missed, % median (IQR)	27.50 (0-52)	40 (15-62.50)	32.50 (7.50-57.50)
	Work impairment score, median (IQR)	7 (2.75-10)	7 (3-10)	10 (2.25-10)
	Social impairment score, median (IQR)	3.50 (1-5.75)	3.50 (2-7)	6 (2.75-8)
Satisfaction score, median (IQR)		49.50 (42.50-53.75)	53 (50-59)	52 (47.50-55)

^a^TECCU: Telemonitoring of Crohn’s Disease and Ulcerative Colitis.

^b^IQR: interquartile range.

^c^IBDQ-9: Inflammatory Bowel Disease Questionnaire 9.

^d^EQ-5D: EuroQol 5 dimensions questionnaire.

### Effects

#### Disease Activity

In terms of the effect on disease activity, the proportion of patients in clinical remission at baseline was 48% (10/21) in G_TECCU, 38% (8/21) in G_NT, and 57% (12/21) among the controls. The proportion of patients in remission improved after 24 weeks in all 3 groups, although this improvement was stronger among the G_TECCU participants, even after considering in the likelihood-based analysis the 3 patients who did not complete the follow-up schedule in G_TECCU: 81% (17/21) of G_TECCU patients were in remission as opposed to 67% (14/21) in the G_NT and 71% (15/21) in the G_control. The proportion of patients in remission increased by 0.33 in G_TECCU, 0.29 in G_NT, and 0.14 in G_control. Thus, the incremental efficacy of G_TECCU was 0.33–0.14=0.19 relative to G_control (median incremental efficacy calculated with the bootstrapping procedure was 0.21, 95% CI –0.07 to 0.66), and the incremental efficacy of TECCU was 0.33–0.29=0.04 relative to the G_NT (median incremental efficacy calculated with the bootstrapping procedure was 0.06, 95% CI –0.16 to 0.43).

#### Quality of Life

The median EQ-5D score improved after 24 weeks in all 3 arms, from 0.816 to 1.00 in G_control and from 0.825 to 1.00 in G_NT and G_TECCU (overall intervention effect on the EQ-5D score: odds ratio 1.99, 95% CI 1.09-3.63; *P*<.001). Consequently, the improvement in the median EQ-5D score in the control group was 0.184 (ie, 1.00–0.816), and that in the G_NT and G_TECCU was 0.175 (ie, 1.00–0.825). Considering that participant mortality was zero and given the 24-week (6-month) time frame of this study, the number of QALYs gained by each patient treated with the standard intervention (controls) was 0.184×(6/12)=0.092 QALYs, and for each patient treated in G_NT and G_TECCU it was 0.175×(6/12)=0.088 QALYs.

All patients considered the care received through the telemonitoring platform to be useful. The 89% (16/18) surveyed patients in G_TECCU indicated that the distance follow-up took little time. The 94% (17/18) surveyed patients rated the quality of the services received remotely as high (8 or more out of 10 points), which helped them to more effectively face their disease-related problems. In all 3 groups, satisfaction scores were high at baseline and at 24 weeks. According to the adapted version of the Client Satisfaction Questionnaire, with a maximum score of 60 points, patient satisfaction improved from a median score of 52 to 57 in G_TECCU and from 49.50 to 55 in G_control (overall intervention effect: odds ratio 8.93, 95% CI 2.97-26.84; *P*<.001) at 24 weeks. However, the satisfaction score remained unchanged at 53 points in G_NT.

### Costs

We calculated the costs per unit of health care resources, equipment, and work productivity and social activities (Table 2). The mean costs and utilities per patient, as well as the bootstrapped differences, were compared between G_TECCU vs G_control (Tables 3 and 4), G_TECCU vs G_NT (Tables 5 and 6), and G_NT vs G_control (Tables 7 and 8).

**Table 2:** Costs per unit of health care resources, equipment, and productivity and social activities.

Costs		Unit	Cost per unit, € (US $)
**Health care costs**			
	Emergency room visits	Visit	189 (206.89)
	Outpatient visits	Visit	40.02 (43.81)
	Hospitalization	Day	310.17 (339.53)
	Hospitalization due to surgical intervention	Day	378 (413.78)
	Telephone calls	Contact	15 (16.42) nurse 21.47 (23.50) physician
	Cost of TECCU^a^ controls	Contact	1.68 (1.84) nurse 3.04 (3.33) physician
**Equipment costs**			
	TECCU rental costs	Patient/month	3.99 (4.37)
**Productivity costs**			
	Work absenteeism (sick leave)	Hour	12.04-25.23 (13.18-27.62)
	Work presenteeism (due to disease activity)	Hour	12.04-25.23 (13.18-27.62)
	Absenteeism for medical visit	Hour	12.04-25.23 (13.18-27.62)
	Absenteeism for telephone call	Hour	12.04-25.23 (13.18-27.62)
	Leisure time used in TECCU contacts	Hour	9.18 (10.05)

^a^TECCU: Telemonitoring of Crohn’s Disease and Ulcerative Colitis.

**Table 3:** Costs per patient in the group receiving remote monitoring (G_TECCU) vs standard care (G_control).

Costs		TECCU^a^ (n=21), mean (SD)		Controls (n=21), mean (SD)		Bootstrapped difference in costs (rounded values), € (US $)	
		Number of units	Cost per patient, €; US $	Number of units	Cost per patient, €; US $	Median	95% CI
**Health care costs**							
	Emergency room visits and nonscheduled outpatient visits	0.27 (0.68)	51.03 (115.06); 55.86 (125.95)	0.19 (0.87)	36.18 (165.03); 39.60 (180.65)	17 (19)	–57 to 116 (62 to 127)
	Outpatient visits	3.79 (1.38)	151.67 (58.72); 166.03 (64.28)	6.30 (0.57)	252.13 (22.91); 276.00 (25.08)	–96 (–105)	–126 to –65 (–138 to –71)
	Hospitalization	0.11 (0.32)	32.63 (97.70); 35.72 (106.95)	0.05 (0.22)	14.79 (67.65); 16.19 (74.05)	16 (18)	–29 to 63 (–32 to 69)
	Hospitalization due to surgical intervention	0.19 (0.89)	71.82 (312.89); 78.62 (342.51)	0.17 (0.87)	65.10 (297.74); 71.26 (325.93)	3 (3)	–195 to 205 (–213 to 224)
	Telephone calls	0.51 (0.76)	9.18 (11.31); 10.05 (12.38)	2.04 (1.62)	32.05 (21.41); 35.08 (23.44)	–24 (–26)	–33 to –14 (–36 to –15)
	Cost of TECCU controls	33.15 (5.78)	68.96 (12.07); 75.49 (13.21)	N/A^b^	N/A	67 (73)	59 to 75 (65 to 82)
**Equipment costs**							
	TECCU rental costs	6 months	23.94 (0); 26.21 (0)	N/A	N/A	24 (26)	22 to 27 (24 to 30)
**Productivity costs**							
	Work absenteeism (sick leave)	15.09 (21.19)	213.97 (300.51); 234.23 (328.96)	25.77 (27.25)	384.22 (418.00); 420.59 (457.57)	–42 (–46)	–234 to 146 (–256 to 160)
	Work presenteeism (due to disease activity)	11.88 (17.26)	168.42 (224.04); 184.36 (245.25)	24.35 (34.53)	363.06 (549.48); 397.43 (601.50)	–89 (–97)	–299 to 91 (–327 to 100)
	Absenteeism for medical visits	13.20 (4.93)	187.22 (62.30); 204.94 (68.20)	20.79 (4.79)	309.98 (113.41); 339.32 (124.15)	–125 (–137)	–189 to –58 (–207 to –63)
	Absenteeism for telephone calls	0.12 (0.15)	1.71 (2.24); 1.87 (2.45)	0.36 (0.27)	5.39 (3.94); 5.90 (4.31)	–3.81 (–4.17)	–5.82 to –2 (–6.37 to –2.19)
	Leisure time used in TECCU contacts	1.79 (0.31)	25.37 (4.42); 27.77 (4.84)	N/A	N/A	25 (27)	22 to 29 (24 to 32)
Total productivity costs per patient (rounded values)		N/A	407 (339); 445 (371)	N/A	678 (686); 742 (751)	–260 (–285)	–600 to 71 (–657 to 78)
Total costs per patient (rounded values)		N/A	807 (623); 883 (682)	N/A	1066 (678); 1167 (742)	–211 (–231)	–600 to 180 (–657 to 197)

^a^TECCU: Telemonitoring of Crohn’s Disease and Ulcerative Colitis.

^b^N/A: not applicable.

**Table 4:** Utilities per patient in the group receiving remote monitoring (G_TECCU) vs standard care (G_control).

Utilities		TECCU^a^ (n=21)	Controls (n=21)	Bootstrapped difference in effects (rounded values)	
				Median	95% CI
**Effects**					
	EQ-5D^b^ score week 24, mean (SD)	0.90 (0.19)	0.93 (0.15)	–0.03	–0.14 to 0.08
	EQ-5D score improvement week 0-24, mean (SD)	0.09 (0.28)	0.10 (0.19)	–0.02	–0.16 to 0.11
	Weeks in remission, mean (SD)	17.89 (7.03)	14.27 (8.13)	3.87	–1.09 to 8.72
	Calprotectin at week 24 (μg/g), median (interquartile range)	126 (47.24)	230 (48.67)	–104	–504 to 75
	Remission at week 24, n (%)	17 (80.95)	15 (71.43)	0.19	–0.01 to 0.42
	Improvement in remission week 0-24, n (%)	7 (33.33)	3 (14.28)	0.21	–0.07 to 0.66
ICER^c^ remission response (rounded values), € (US $)		N/A^d^	N/A	–1005 (–1100)	–13,518 to 3137 (–14,798 to 3434)
ICER quality-adjusted life-years (rounded values), € (US $)		N/A	N/A	9078 (9937)	–56,547 to 44,628 (–61,900 to 48,853)

^a^TECCU: Telemonitoring of Crohn’s Disease and Ulcerative Colitis.

^b^EQ-5D: EuroQol 5 dimensions questionnaire.

^c^ICER: incremental cost-effectiveness ratio.

^d^N/A: not applicable.

**Table 5:** Costs per patient in the group receiving remote monitoring (G_TECCU) vs nurse-assisted telephone care (G_NT).

Costs		TECCU^a^ (n=21), mean (SD)		Telephone care (n=21), mean (SD)		Bootstrapped difference in costs (rounded values), € (US $)	
		Number of units	Cost per patient, €; US $	Number of units	Cost per patient, €; US $	Median	95% CI
**Health care costs**							
	Emergency room visits and nonscheduled outpatient visits	0.27 (0.68)	51.03 (115.06); 55.86 (125.95)	0.57 (1.17)	108.15 (220.18); 118.39 (241.02)	–48 (–53)	–150 to 55 (–164 to 60)
	Outpatient visits	3.79 (1.38)	151.67 (58.72); 166.03 (64.28)	4.25 (1.92)	170.03 (76.68); 186.13 (83.94)	–13 (–14)	–59 to 30 (–65 to 33)
	Hospitalization	0.11 (0.32)	32.63 (97.70); 35.72 (106.95)	0.24 (0.77)	73.83 (238.20); 80.82 (260.75)	–43 (–47)	–161 to 48 (–176 to 53)
	Hospitalization due to surgical intervention	0.19 (0.89)	71.82 (312.89); 78.62 (342.51)	0.26 (1.31)	97.41 (446.49); 106.63 (488.76)	–29 (–32)	–292 to 204 (–320 to 223)
	Telephone calls	0.51 (0.76)	9.18 (11.31); 10.05 (12.38)	5.27 (1.35)	85.03 (20.31); 93.08 (22.23)	–76 (–83)	–85 to –66 (–93 to –72)
	Cost of TECCU controls	33.15 (5.78)	68.96 (12.07); 75.49 (13.21)	N/A^b^	N/A	67 (73)	59 to 75 (65 to 82)
**Equipment costs**							
	TECCU rental costs	6 months	23.94 (0); 26.21 (0)	N/A	N/A	24 (26)	22 to 27 (24 to 30)
**Productivity costs**							
	Work absenteeism (sick leave)	15.09 (21.19)	213.97 (300.51); 234.23 (328.96)	13.52 (12.51)	206.74 (194.61); 226.31 (213.03)	30 (33)	–106 to 185 (–116 to 203)
	Work presenteeism (due to disease activity)	11.88 (17.26)	168.42 (224.04); 184.36 (245.25)	11.58 (20.53)	177.09 (317.12); 193.85 (347.14)	–40 (–44)	–186 to 102 (–204 to 112)
	Absenteeism for medical visits	13.20 (4.93)	187.22 (62.30); 204.94 (68.20)	14.03 (6.32)	214.52 (91.72); 234.83 (100.40)	–27 (–30)	–93 to –36 (–102 to –39)
	Absenteeism for telephone calls	0.12 (0.15)	1.71 (2.24); 1.87 (2.45)	1.06 (0.31)	15.69 (4.59); 17.18 (5.02)	–14 (–15)	–16 to –12 (–18 to –13)
	Leisure time used in TECCU contacts	1.79 (0.31)	25.37 (4.42); 27.77 (4.84)	N/A	N/A	25 (27)	22 to 27 (24 to 30)
Total productivity costs per patient (rounded values)		N/A	407 (339); 445 (371)	N/A	466 (397); 510 (435)	–51 (–56)	–292 to 199 (–320 to 218)
Total costs per patient (rounded values)		N/A	807 (623); 883 (682)	N/A	992 (804); 1086 (880)	–135 (–148)	–579 to 290 (–634 to 317)

^a^TECCU: Telemonitoring of Crohn’s Disease and Ulcerative Colitis.

^b^N/A: not applicable.

**Table 6:** Utilities per patient in the group receiving remote monitoring (G_TECCU) vs nurse-assisted telephone care (G_NT).

Utilities			TECCU^a^ (n=21)		Telephone care (n=21)		Bootstrapped difference in effects (rounded values)		
							Median		95% CI
**Effects**									
	EQ-5D^b^score week 24, mean (SD)	0.90 (0.19)		0.89 (0.16)		0.01		–0.10 to 0.12	
	EQ-5D score improvement week 0-24, mean (SD)	0.09 (0.28)		0.08 (0.18)		–0.01		–0.15 to 0.13	
	Weeks in remission, mean (SD)	17.89 (7.03)		17.24 (8.38)		1.62		–3.53 to 6.67	
	Calprotectin at week 24 (μg/g), median (interquartile range)	126 (47.24)		168 (49.38)		–12.31		–167 to 133	
	Remission at week 24, n (%)	17 (80.95)		14 (66.67)		0.17		–0.01 to 0.48	
	Improvement in remission week 0-24, n (%)	7 (33.33)		6 (28.57)		0.06		–0.16 to 0.43	
ICER^c^ remission response (rounded values), € (US $)			N/A^d^		N/A		–2250 (–2463)		–15,363 to 11,086 (–16,817 to 12,135)
ICER quality-adjusted life-years (rounded values), € (US $)			N/A		N/A		5761 (6306)		–36,109 to 47,231 (–39,527 to 51,702)

^a^TECCU: Telemonitoring of Crohn’s Disease and Ulcerative Colitis.

^b^EQ-5D: EuroQol 5 dimensions questionnaire.

^c^ICER: incremental cost-effectiveness ratio.

^d^N/A: not applicable.

**Table 7:** Costs per patient in the group receiving nurse-assisted telephone care (G_NT) vs standard care (G_control).

Costs		Telephone care (n=21), mean (SD)		Controls (n=21), mean (SD)		Bootstrapped difference in costs (rounded values), € (US $)		
		Number of units	Cost per patient, €; US $	Number of units	Cost per patient, €; US $		Median	95% CI
**Health care costs**								
	Emergency room visits and nonscheduled outpatient visits	0.57 (1.17)	108.15 (220.18); 118.39 (241.02)	0.19 (0.87)	36.18 (165.03); 39.60 (180.65)		72 (79)	–45 to 180 (–49 to 197)
	Outpatient visits	4.25 (1.92)	170.03 (76.68); 186.13 (83.94)	6.30 (0.57)	252.13 (22.91); 276.00 (25.08)		–84 (–92)	–119 to –39 (–130 to –43)
	Hospitalization	0.24 (0.77)	73.83 (238.20); 80.82 (260.75)	0.05 (0.22)	14.79 (67.65); 16.19 (74.05)		59 (65)	–29 to 177 (–32 to 194)
	Hospitalization due to surgical intervention	0.26 (1.31)	97.41 (446.49); 106.63 (488.76)	0.17 (0.87)	65.10 (297.74); 71.26 (325.93)		32 (35)	–195 to 292 (–213 to 320)
	Telephone calls	5.27 (1.35)	85.03 (20.31); 93.08 (22.23)	2.04 (1.62)	32.05 (21.41); 35.08 (23.44)		52 (57)	40 to 64 (44 to 70)
	Cost of TECCU^a^ controls	N/A^b^	N/A	N/A	N/A		N/A	N/A
**Equipment costs**								
	TECCU rental costs	N/A	N/A	N/A	N/A		N/A	N/A
**Productivity costs**								
	Work absenteeism (sick leave)	13.52 (12.51)	206.74 (194.61); 226.31 (213.03)	25.77 (27.25)	384.22 (418.00); 420.59 (457.57)		–72 (79)	–247 to 79 (–270 to 86)
	Work presenteeism (due to disease activity)	11.58 (20.53)	177.09 (317.12); 193.85 (347.14)	24.35 (34.53)	363.06 (549.48); 397.43 (601.50)		–49 (–54)	–286 to 143 (–313 to 157)
	Absenteeism for medical visits	14.03 (6.32)	214.52 (91.72); 234.83 (100.40)	20.79 (4.79)	309.98 (113.41); 339.32 (124.15)		–98 (–107)	–173 to –18 (–189 to –20)
	Absenteeism for telephone calls	1.06 (0.31)	15.69 (4.59); 17.18 (5.02)	0.36 (0.27)	5.39 (3.94); 5.90 (4.31)		10 (11)	8 to 13 (9 to 14)
	Leisure time used in TECCU contacts	N/A	N/A	N/A	N/A		N/A	N/A
Total productivity costs per patient (rounded values)		N/A	466 (397); 510 (435)	N/A	678 (686); 742 (751)		–209 (–229)	–570 to 125 (–624 to 137)
Total costs per patient (rounded values)		N/A	992 (804); 1086 (880)	N/A	1066 (678); 1167 (742)		–77 (–84)	–524 to 378 (–574 to 414)

^a^TECCU: Telemonitoring of Crohn’s Disease and Ulcerative Colitis.

^b^N/A: not applicable.

**Table 8:** Utilities per patient in the group receiving nurse-assisted telephone care (G_NT) vs standard care (G_control).

Utilities		Telephone care (n=21)	Controls (n=21)	Bootstrapped difference in effects (rounded values)	
				Median	95% CI
**Effects**					
	EQ-5D^a^ score week 24, mean (SD)	0.89 (0.16)	0.93 (0.15)	–0.04	–0.13 to 0.05
	EQ-5D score improvement week 0-24, mean (SD)	0.08 (0.18)	0.10 (0.19)	–0.02	–0.11 to 0.09
	Weeks in remission, mean (SD)	17.24 (8.38)	14.27 (8.13)	2.28	–2.38 to 7.33
	Calprotectin at week 24 (μg/g), median (interquartile range)	168 (49.38)	230 (48.67)	–91	–505 to 117
	Remission at week 24, n (%)	14 (66.67)	15 (71.43)	–0.05	–0.33 to 0.24
	Improvement in remission week 0-24, n (%)	6 (28.57)	3 (14.28)	0.14	–0.19 to 0.48
ICER^b^ remission response (rounded values), € (US $)		N/A^c^	N/A	–538 (–589)	–6475 to 5303 (–7088 to 5805)
ICER quality-adjusted life-years (rounded values), € (US $)		N/A	N/A	3316 (3630)	–58,652 to 35,482 (–64,204 to 38,841)

^a^EQ-5D: EuroQol 5 dimensions questionnaire.

^b^ICER: incremental cost-effectiveness ratio.

^c^N/A: not applicable.

#### G_TECCU Versus G_control

After the 24-week follow-up, the total mean cost per G_TECCU patient was €807 (US $883) as opposed to €1066 (US $1167) for the control patients, representing a median cost reduction from a societal perspective of €211 (US $231) per patient (95% CI €–600 to 180 per patient; US $–657 to 197 per patient). The main drivers of health care costs were the reduction of €96 (US $105) per patient in outpatient visits (95% CI €–126 to –65; US $–138 to –71) and that of €24 (US $26) per patient in telephone consultations (95% CI €–33 to –14; US $–36 to –15). Productivity costs were reduced in the G_TECCU intervention group by €260 (US $285) per patient (95% CI €–600 to 71; US $–657 to 78). Reduced absenteeism due to outpatient visits (€–125 per patient, 95% CI €–189 to –58; US $–137 per patient, 95% CI US $–207 to –63) and telephone consultations (€–3.81 per patient, 95% CI €–5.82 to –2; US $–4.17 per patient, 95% CI US $–6.37 to –2.19) represented a significant cost saving (see Table 3).

#### G_TECCU Versus G_NT

At 24 weeks, the total mean cost per patient in the G_NT was €992 (US $1086) as opposed to €807 (US $883) for G_TECCU patients. Thus, there was a median cost reduction of €135 (US $148) per patient in the G_TECCU after 24 weeks (95% CI €–579 to 290 per patient; US $–634 to 317 per patient), which was associated with several health care and work productivity factors. The reduction of costs was significant in terms of telephone consultations (€–76 per patient, 95% CI €–85 to –66; US $–83 per patient, 95% CI US $–93 to –72) and the associated absenteeism to attend to those telephone calls (€–14 per patient, 95% CI €–16 to –12; US $–15 per patient, 95% CI US $–18 to –13; see Table 5).

#### G_NT Versus G_control

The mean costs per patient in the G_control and G_NT represented a median cost reduction of €77 (US $84) per patient in G_NT after 24 weeks (95% CI €–524 to 378 per patient; US $–574 to 414 per patient). This costs saving was mainly due to the reduction of €84 (US $92) per patient linked to fewer outpatient visits by patients in G_NT (95% CI €–119 to –39; US $–130 to –43) and a significant reduction of €98 (US $107) per patient for absenteeism due to medical visits (95% CI €–173 to –18; US $–189 to 20; see Table 7).

### Cost-Effectiveness Analysis

#### G_TECCU Versus G_control

We obtained the mean ICER for TECCU care compared with standard care by dividing the incremental costs by the differences in the increment for the percentage of patients in remission. For TECCU, this was €–211/0.191=€–1105 (US $1210) for 1 additional patient in remission in G_TECCU, 24 weeks after inclusion. The bootstrapping procedure gave an estimated median ICER of €–1005 (95% CI €–13,518 to 3137; US $1100, 95% CI US $–14,798 to 3434).

**Figure 3:**
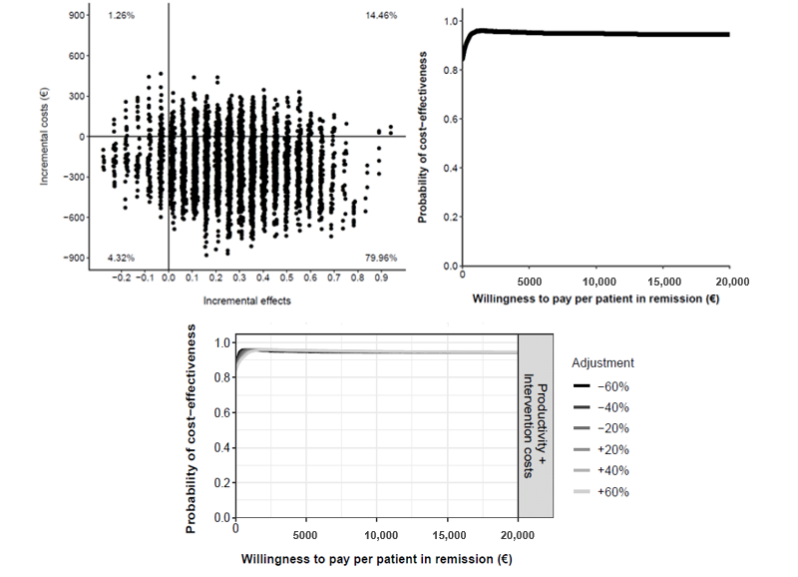
Cost-effectiveness plane (top left) and cost-effectiveness acceptability curve (top right) comparing the Telemonitoring of Crohn’s Disease and Ulcerative Colitis (TECCU) intervention versus standard care. Bottom: impact of different cost values on the cost-effectiveness acceptability curves.

In the cost-effectiveness plane (Figure 3), we represented all the estimated ICERs with dots, and there was a 79.96% probability that TECCU improved the proportion of patients in remission at a lower societal cost than for the control patients (dominant quadrant). In an additional 14.46% of simulations, TECCU produced stronger effects but with higher costs than for the standard caare. The probability that TECCU was cost-effective in comparison with standard care at a WTP of €20,000 (US $21,893) per additional patient in remission was 95%, and the probability that TECCU was cost saving at a WTP of €0 was 84%. These percentages remained stable even after adjusting both the health care (including equipment costs) and indirect costs over a range of ±60%, and the different cost-effectiveness acceptability curves calculated were very similar (Figure 3).

#### G_TECCU Versus G_NT

Considering that TECCU saved €135 per patient relative to G_NT and the difference in the efficacy on disease activity between these 2 interventions was 0.048, the mean ICER of G_TECCU relative to G_NT was € *–* 2812 (€–135/0.048) after 24 weeks. This means that when TECCU achieved 1 additional patient in remission compared with G_NT, the cost savings was €2812 (US $3078). Using the bootstrapping procedure we estimated that the median ICER was €–2250 (95% CI €–15,363 to 11,086; US $–2463, 95% CI US $–16,817 to 12,135).

**Figure 4:**
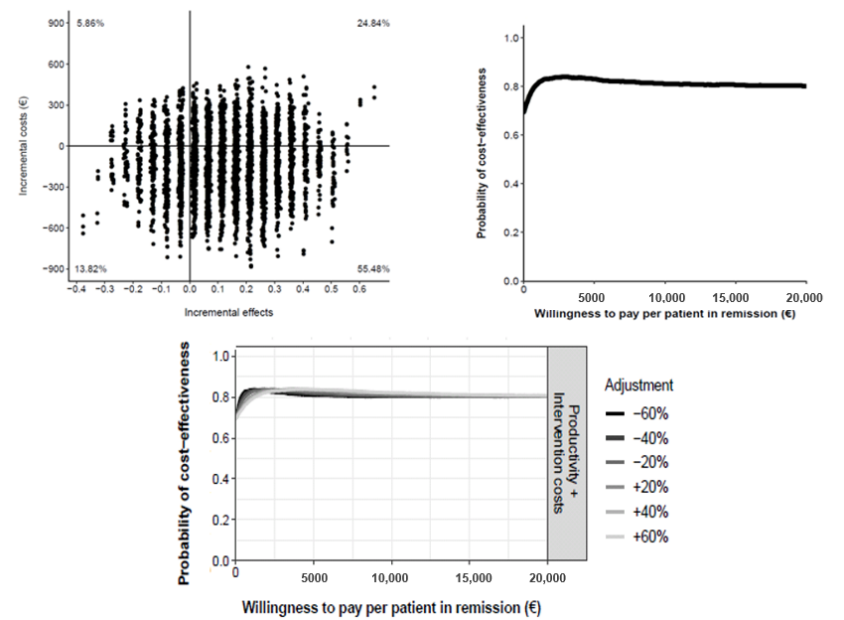
Cost-effectiveness plane (top left) and cost-effectiveness acceptability curve (top right) comparing Telemonitoring of Crohn’s Disease and Ulcerative Colitis (TECCU) versus telephone care. Bottom: impact of different cost values on the cost-effectiveness acceptability curves.

In the cost-effectiveness plane (Figure 4), we found that 55.48% of the dots fell in the dominant quadrant, indicating that this was the probability of TECCU being more effective than telephone care at a lower societal cost. In another 24.84% of the simulations, TECCU had a stronger effect but higher costs than telephone care. The probability that TECCU was cost-effective relative to G_NT at a WTP of €20,000 (US $21,893) per additional patient in remission was 81%, and the probability that TECCU was more cost saving at a WTP of €0 was 69%. In all sensitivity scenarios (±60%), the probability of TECCU being cost-effective at a WTP of €0 (cost saving) was 69%, and this probability increased to a stable 80% to 81% at a WTP of €20,000 or more (Figure 4).

#### G_NT Versus G_control

Comparing the mean ICER of telephone care with that of standard care, by dividing the incremental costs by the differences in the increase of the percentage of patients in remission, it showed a cost of €–77/0.143= €–538 (US $–589) for 1 additional patient in remission 24 weeks after inclusion. Using the bootstrapping procedure, we estimated that the median ICER was €–538 (95% CI €–6475 to 5303; US $–589, 95% CI US $–7088 to 5805).

**Figure 5:**
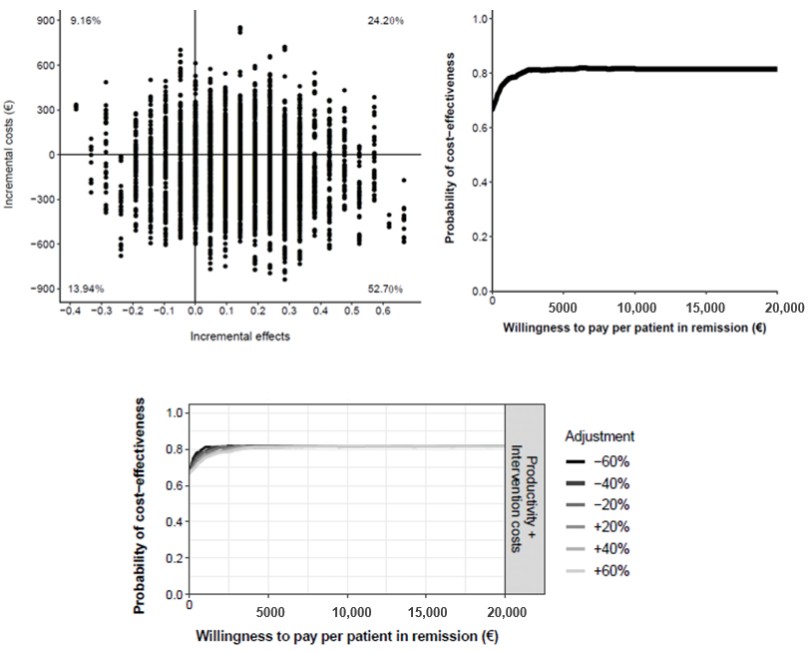
Cost-effectiveness plane (top left) and cost-effectiveness acceptability curve (top right) comparing telephone care versus standard care. Bottom: impact of different cost values on the cost-effectiveness acceptability curves.

In the cost-effectiveness plane (Figure 5), 52.70% fell into the dominant quadrant, indicating that there was a 52.70% probability that nurse-assisted telephone care was more effective than standard care at a lower societal cost. In 24.20% of the simulations, telephone care was more effective than standard care at a higher societal cost. The probability that telephone care was cost saving relative to standard care was 67% at a WTP of €0 per additional patient in remission, whereas at a WTP of €20,000 (US $21,893) the probability of telephone care being cost-effective was 81%. After modifying the costs over a range of ±60%, the probability of telephone care being more cost saving remained unchanged at 67% at a WTP of €0. The probability that telephone care was more cost-effective at a WTP of €20,000 or more was stable at 81% (Figure 5).

### Cost-Utility Analysis

#### G_TECCU Versus G_control

The baseline EQ-5D scores were slightly higher in G_TECCU and G_NT, and thus the improvement over the 24-week follow-up was not greater than that in G_control. Using the bootstrapping procedure, the median ICER for 1 additional QALY was estimated to be €9078 (95% CI €–56,547 to 44,628; US $9937, 95% CI US $–61,900 to 48,853), which means that €9078 was saved in G-TECCU for each extra QALY gained in the G_control.

**Figure 6:**
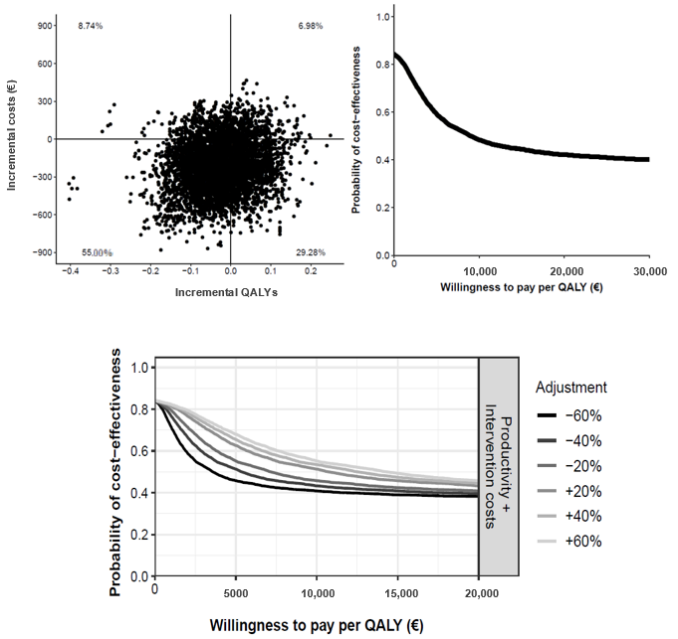
Cost-effectiveness plane (top left) and cost-effectiveness acceptability curve (top right) comparing the effect on quality-adjusted life-years (QALYs) of Telemonitoring of Crohn’s Disease and Ulcerative Colitis (TECCU) versus standard care. Bottom: impact of different cost values on the cost-utility acceptability curves.

There was a 29.28% probability that the TECCU intervention was associated with a higher gain in QALYs at a lower cost (Figure 6). In another 55.00% of the simulations, TECCU was still less expensive than standard care, although with a weaker improvement in QALYs gained. Thus, considering the statistical uncertainty of the costs and QALYs calculated, about 84% of the bootstrapped ICERs were associated with cost savings, which is the probability of TECCU being cost-effective relative to standard care at a WTP of €0. However, the cost-effectiveness acceptability curve was a decreasing function of WTP because approximately 64% of simulations did not involve health gains [43]. Thus, the probability of TECCU being cost-effective at a WTP of €20,000 (US $21,893) fell to 42%, and this probability remained stable (40% to 45%) after modifying the costs in the sensitivity analysis (Figure 6).

#### G_TECCU Versus G_NT

The median cost-utility ratio per QALY gained with TECCU relative to telephone care was €5761 (95% CI €–36,109 to 47,231; US $6306, 95% CI US $–39,527 to 51,702), meaning that €5761 was saved in G_TECCU for each extra QALY gained in the G_NT.

**Figure 7:**
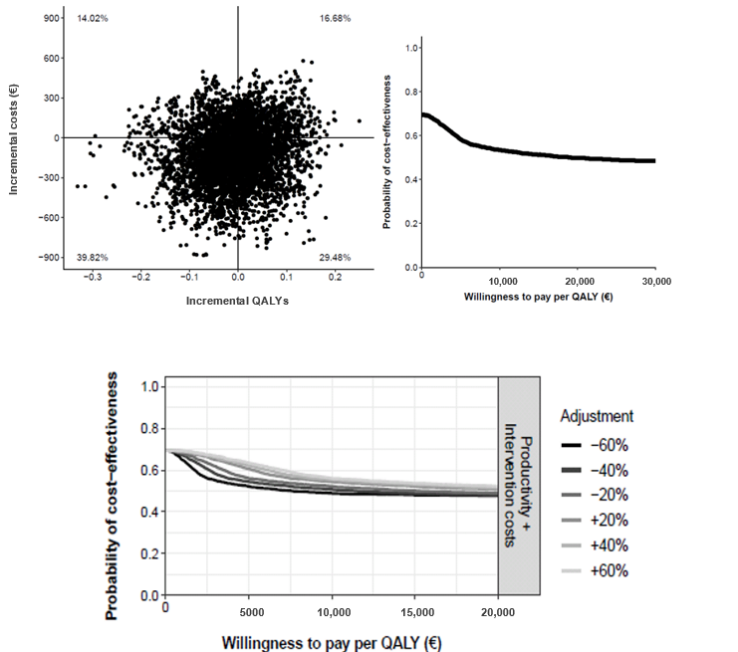
Cost-effectiveness plane (top left) and cost-effectiveness acceptability curve (top right) comparing the effect on quality-adjusted life-years (QALYs) of Telemonitoring of Crohn’s Disease and Ulcerative Colitis (TECCU) versus telephone care. Bottom: impact of different cost values on the cost-utility acceptability curves.

The probability that TECCU led to a higher QALY gain at lower costs was 29.48% (Figure 7), while in another 39.82% of the simulations TECCU was still associated with lower costs but with a weaker improvement in QALYs gained. Considering statistical uncertainty, the cost-effectiveness acceptability curve suggested a 69% probability that TECCU was more cost saving at a WTP of €0 than telephone care in terms of QALYs. However, as approximately 54% of the simulations did not involve health gains, the probability of TECCU being cost-effective at a WTP of €20,000 (US $21,893) fell to 50%. In the different cost scenarios calculated in the sensitivity analysis, the range of this probability was tight (48% to 51%; Figure 7).

#### G_NT Versus G_control

The median incremental societal costs per QALY gained in the G_NT relative to the controls was €3316 (95% CI €–58,652 to 35,482; US $3630, 95% CI US $–64,204 to 38,841), which means that telephone care saved €3316 for each additional QALY gained in the G_control.

**Figure 8:**
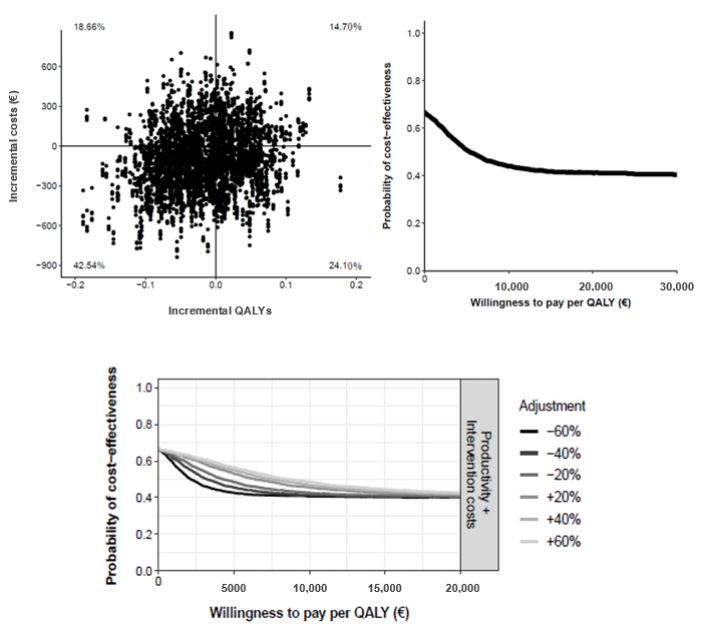
Cost-effectiveness plane (top left) and cost-effectiveness acceptability curve (top right) comparing the effect on quality-adjusted life-years (QALYs) of telephone care versus standard care. Bottom: impact of different cost values on the cost-utility acceptability curves.

There was a 24.10% probability that nurse-assisted telephone care was associated with a higher QALY gain at lower costs (Figure 8). In a further 42.54% of simulations, telephone care was less expensive than standard care but with a lower improvement in QALYs gained. With the combination of these percentages, approximately 67% of simulations estimated that telephone care was less costly than standard care (cost-effective at a WTP €0). However, as about 61% of simulations did not involve health gains, the probability that telephone care was cost-effective fell to 41% at a WTP of €20,000 (US $21,893). Considering scenarios in which health care and indirect costs decrease or increase over 60%, at a WTP of €20,000 the probability of telephone care being cost-effective fell to 40% and 42%, respectively (Figure 8).

## Discussion

### Principal Findings

To the best of our knowledge, this is the first study to evaluate the cost-effectiveness and cost-utility of a Web telemonitoring platform for IBD patients from a societal perspective. We used a standard methodology [21] that examined both health care and non–health care–related costs against the effect of the TECCU Web platform on health outcomes, and to compare this with telephone care and standard care, the main strategies currently used for the follow-up of IBD patients. This economic evaluation included topics and outcome measures recommended in the model for assessment of telemedicine [44]. Other approaches such as the monitoring and assessment framework for the European Innovation Partnership are available to estimate the health and economic outcomes of eHealth interventions [45]. In any case, as the 24-week period after initiation of immunosuppressants or biologic agents may be different from the maintenance therapy period, we avoided performing economic estimations with longer time horizons.

In this regard, TECCU had a stronger effect on disease activity, with associated savings of €1005 (US $1100) and €2250 (US $2463) for each additional patient in remission when compared with standard care and telephone care, respectively. Conversely, while quality-of-life scores improved in all 3 groups, neither TECCU nor telephone care produced a stronger improvement than standard care, possibly due to the higher baseline scores of G_TECCU and G_NT patients, and to the relatively small sample size, hindering the possibility of detecting statistical differences after 24 weeks. However, TECCU and telephone care were associated with an 84% and 67% probability, respectively, of reducing costs per additional QALY relative to the controls.

The WTP threshold that can be considered acceptable to consider an intervention cost-effective is not clear, and in Spain it was recently estimated to lie between €22,000 (US $24,082) and €25,000 (US $27,367) per QALY [46]. Far from these values, the probability of TECCU being more effective than standard care was 80%, even at a lower societal cost. Furthermore, the probability of being cost-effective was 95% at a WTP of €20,000 (US $21,893) per additional patient in remission. The differences with respect to telephone care were lower, although comparing these interventions there was still an 81% likelihood that telemonitoring was cost-effective at a WTP of €20,000 per additional patient in remission. Moreover, all these probabilities were stable after considering alternative costing scenarios.

While we evaluated the cost-effectiveness and cost-utility of the TECCU Web platform, economics data regarding eHealth interventions in IBD have been scarce thus far. This represents a barrier to their implementation in real life because they are associated with a good cost-effectiveness profile in some chronic pathologies such as cardiovascular diseases [47], but not in others [48]. Considering IBD, 2 clinical trials previously assessed a Web-based approach to guide patient self-treatment, demonstrating a significant reduction in direct costs by replacing outpatient visits with distance care [12,49]. A remote management program developed in the United Kingdom for patients with stable IBD also estimated that virtual clinics could potentially save £119,000 (US $143,072) per year [14]. However, these savings analyses did not consider indirect costs, and a reduction in travel time was only described in an uncontrolled pilot trial of patients who used telehealth [50]. It is surprising that even if the use of telemonitoring for IBD is associated with a shift from in-person visits toward remote encounters [12,13,16], previous studies did not include costs associated with the purchase of the necessary equipment and with remote contacts.

The use of TECCU was associated with a median saving of €211 (US $231) per patient relative to standard care. Considering health care costs, TECCU saved €94 (US $103) per patient in outpatient visits and €24 (US $26) per patient in telephone calls, these savings in outpatient visits over 6 months being very similar to the €189 (US $207) per patient per year reported previously in a clinical trial with an eHealth program [12]. However, the median expense of €71 (US $78) per patient in telemonitoring contacts calculated here is comparable with these savings. Thus, the median of €211 (US $231) saved per patient from a societal perspective is mainly associated with the improvement in work productivity and not with the benefits in health care costs, as reported in previous studies where expenditure in telemonitoring was not considered. By contrast, when compared with another distance follow-up method such as telephone care, TECCU cost savings were related to both health care and non–health care costs.

### Strengths and Limitations

The strengths of this study include the use of validated clinical indexes to assess the effects of the 3 follow-up methods on disease activity and quality of life. We considered national and regional official prices to calculate costs and we chose the societal perspective to perform the economic evaluation in this study, including costs associated with health care and investment in equipment, and costs related to patients’ productivity at work and social activity impairment in all 3 groups. Additionally, to characterize the uncertainty of the costs and utilities calculated, we used nonparametric bootstrap estimations, as well as a sensitivity analysis to examine whether the ICERs changed in alternative costing scenarios. Finally, to better reproduce the costs calculated in a real-world setting, the follow-up schedule for all 3 arms in our clinical trial was designed according to the standard clinical practice in our center and based on national and European guidelines, as published elsewhere [20].

This study had a series of limitations. First, quality of life was a secondary outcome, and we only measured it at baseline and at the end of the study to improve adherence to the follow-up schedule. This limitation, associated with the reduced sample size and the higher baseline scores in G_TECCU and G_NT, could hinder the possibility to detect significant differences after 24 weeks. Second, even though we recruited patients consecutively in a referral center, the sample size was relatively small, mainly because we only included patients with complex IBD during the initiation of immunosuppressants or biologic agents, but not those on maintenance therapy. Although we used validated questionnaires to measure the effects and the official rates for Spain, the study of this specific population may compromise the generalization of cost data to other settings. Third, the trial did not consider travel costs related to in-person medical visits, but in any case this would underestimate the cost savings associated with TECCU and telephone care. Fourth, the economic evaluation was limited to the 24 weeks of the study period, as we evaluated patients with complex IBD at the initiation of treatment with immunosuppresants or biologic agents, or both. In this sense, it is possible that costs and effects would change after longer follow-up periods with maintenance therapy, and further studies considering longer time horizons will be necessary.

### Conclusion

There is a high probability that the use of the TECCU Web platform for telemonitoring patients with complex IBD produces a greater improvement in disease activity at a lower societal cost, compared with both standard care and telephone care. Considering the increasing burden and costs of managing IBD worldwide, as well as the lack of economic data related to eHealth interventions, our results provide important information regarding the cost-effectiveness of Web telemonitoring for IBD. The use of systems such as TECCU could be a real option to help reorganize the structure of national health systems in the future. However, further studies are still necessary to evaluate the impact of eHealth on quality of life and its cost-effectiveness in larger sample sizes and over longer periods.

## Appendix

Multimedia Appendix 1 CONSORT-EHEALTH checklist V1.6.1.

## Abbreviations

eHealth: electronic health
EQ-5D: EuroQol 5 dimensions questionnaire
G_control: group receiving standard care with in-person visits
G_NT: group receiving nurse-assisted telephone care
G_TECCU: group receiving remote monitoring
IBD: inflammatory bowel disease
ICER: incremental cost-effectiveness ratio
QALY: quality-adjusted life-year
TECCU: Telemonitorización de la Enfermedad de Crohn y Colitis Ulcerosa (Telemonitoring of Crohn’s Disease and Ulcerative Colitis
WTP: willingness to pay
